# Turn-timing in signed conversations: coordinating stroke-to-stroke turn boundaries

**DOI:** 10.3389/fpsyg.2015.00268

**Published:** 2015-03-24

**Authors:** Connie de Vos, Francisco Torreira, Stephen C. Levinson

**Affiliations:** ^1^Language and Cognition Department, Max Planck Institute for Psycholinguistics, NijmegenNetherlands; ^2^Radboud University, NijmegenNetherlands

**Keywords:** turn-taking, turn-timing, visual-gestural modality, sign language, sign phonetics, conversation analysis

## Abstract

In spoken interactions, interlocutors carefully plan, and time their utterances, minimizing gaps and overlaps between consecutive turns. Cross-linguistic comparison has indicated that spoken languages vary only minimally in terms of turn-timing, and language acquisition research has shown pre-linguistic vocal turn-taking in the first half year of life. These observations suggest that the turn-taking system may provide a fundamental basis for our linguistic capacities. The question remains, however, to what extent our capacity for rapid turn-taking is determined by modality constraints. The avoidance of overlapping turns could be motivated by the difficulty of hearing and speaking at the same time. If so, turn-taking in sign might show greater toleration for overlap. Alternatively, signed conversations may show a similar distribution of turn-timing as spoken languages, thus avoiding both gaps and overlaps. To address this question we look at turn-timing in question–answer sequences in spontaneous conversations of Sign Language of the Netherlands. The findings indicate that although there is considerable overlap in two or more signers’ articulators in conversation, when proper allowance is made for onset preparation, post-utterance retraction and the intentional holding of signs for response, turn-taking latencies in sign look remarkably like those reported for spoken language. This is consistent with the possibility that, at least with regard to responses to questions, speakers and signers follow similar time courses in planning and producing their utterances in on-going conversation. This suggests that turn-taking systems may well be a shared cognitive infrastructure underlying all modern human languages, both spoken and signed.

## Introduction

Spontaneous conversations among speakers often run smoothly with slight overlaps and gaps between consecutive turns (Sacks et al., 1974). Comparative research has shown that speakers from a broad range of typologically and geographically dispersed languages vary little in response latencies in question–answer sequences, with mean overall offsets at 229 ms, and language-specific means within 250 ms on either side of this cross-language mean (Stivers et al., 2009). A general observation in studies of spoken interaction is that speakers orient toward a one-at-a-time principle when taking turns at talk, and do so at a surprisingly fast pace across a wide range of spoken languages. The universality of this tightly organized behavior in spoken conversation, as well as its clear precursors in early infancy, make a case for turn-taking constituting an important part of human communicative ethology (Levinson, 2006). A leading question for the research reported here is to what extent sign language users also operate the same turn-taking system as used in spoken languages, especially with regard to turn-timing.

One of the substantial discoveries of the last 50 years is that sign languages show all the properties of full natural languages on all relevant levels of linguistic structure, including, for instance, sublexical structure at the phonological level (Emmorey, 2002; Meier et al., 2002; Sandler and Lillo-Martin, 2006; Channon and van der Hulst, 2011; Berent et al., 2013). However, it has been suggested that, in conversational turn-taking, signers may show greater toleration for overlap due to the characteristics of the visual modality. In signed conversation, visual feedback does not interfere with sign-production, in the same way that auditive feedback does when speaking (Emmorey et al., 2009). This suggests that, if turn-taking was basically motivated by channel limitations (the difficulty of hearing and speaking at the same time), then signed turn-timing should be characterized by a higher proportion of overlapping turns. Additionally, Coates and Sutton-Spence (2001) argue that unlike speakers, signers do not attend to the one-at-a-time principle, and rather form a collaborative floor with their interlocutors, thus having higher degrees of social tolerance for overlap.

Conversation analytic work on sign languages has been sparse, yet early studies of signed conversations have observed some remarkable features of signed interactions. Baker (1977) was the first to note that unlike speakers, sign language users need to ensure visual attention of their interlocutors before they can initiate a turn, hence the use of various types of summonses is more pervasive in signed interaction due to the localized nature of visual information, which requires attentional focus (McIlvenny, 1995). In addition to explicit attention-getting gestures such as waves and taps, this may be done by repeating the initial sign of a turn until recipiency is confirmed through eye contact. The latter strategy appears akin to recycling turn beginnings in spoken conversation, but has the specific purpose of mobilizing recipiency (cf. Schegloff, 1987). In multi-party conversation, multiple signers may self-select for the next turn and thus initiate signing at the same time, although such overlaps may not always be attended to. In addition, there are many other cases of overlapping movement of participants’ articulators. As such it is important to consider, in the case of sign language, whether overlapping signs are in fact attended to as competitive turns by speech act participants (McIlvenny, 1995; McCleary and de Arantes Leite, 2013; Groeber and Pochon-Berger, 2014). In the current study, we consider turns that make relevant a timely and contingent response on behalf of the addressee, namely question and answer sequences.

In spoken conversation, addressees sometimes initiate a response at a possible point of completion while the initial speaker continues his turn, thus resulting in *terminal overlap* (Jefferson, 1986; Schegloff, 2000). Similarly, Baker (1977) observed the phenomenon of *partial overlap* at signed turn transitions when “one interactant’s hand(s) moving toward the position where a sign will be made as the other interactant is making a sign.” However, in considering the comparison between spoken and signed languages, we must bear in mind that the preparation for vocalization in spoken languages is mostly inaccessible to other participants, consisting apart from inbreaths in early motor preparation of the vocal organs (Palo et al., 2014). Therefore, as argued by McCleary and de Arantes Leite (2013), these preparatory movements in sign function on a par with pre-turn inbreaths or other pre-vocal preparation in spoken languages and thus should arguably be excluded from the analysis of the actual exchange of turns. That is to say, it seems reasonable, in order to compare signed, and spoken interactions on equal grounds, to exclude the preparation for signing from timing analysis, as has been the case with the preparation for speaking in the analysis of spoken turn-taking (McCleary and de Arantes Leite, 2013). In our analysis of turn-timing, we differentiate between partial overlaps and possible completion overlaps by taking the start of the initial *stroke* (the ‘content’ part of the manual gesture) as the turn beginning as it most directly reflects the phonological content of a sign.

Overlap at signed turn transitions may also result from turn-final holds, which are typically released as soon as the relevant response has been recognized (Groeber and Pochon-Berger, 2014). Crucially, signers do not orient to these practices as troublesome in conversation, nor do such overlaps get dealt with using designated overlap resolution devices (cf. Schegloff, 2000; McCleary and de Arantes Leite, 2013). We thus hypothesized that in optimizing turn transitions, sign language users focus on the phonological content of signs as represented by the stroke, and disregard early preparatory movements, and the intentional holding of signs for response, as well as post-utterance retraction. In other words, the end of the final stroke appears to most directly parallel the transition relevance place (TRP) at which a contingent response on behalf of the interlocutor becomes relevant (Sacks et al., 1974).

If turn-taking lies deep in our communicational instincts as has been suggested (Levinson, 2006), then it may be expected to follow broadly similar lines regardless of language modality. We therefore test the prediction that in signed conversations, interlocutors attend to stroke-to-stroke turn boundaries. If this were the case, turn-timing in signed interactions as calculated by stroke-to-stroke turn boundaries should be within the same cross-linguistic range as has previously been reported for spoken languages.

To address this question we analyze turn-timing in 190 question–answer sequences captured from spontaneous conversations of Sign Language of the Netherlands Nederlandse Gebarentaal (NGT). Questions–answer sequences provide a particularly well-suited conversational context in which to investigate turn-timing, as questions make due a conditionally relevant and timely response (cf. Stivers et al., 2009). The signs for each question–answer sequence were coded and checked by native signers for onset, lexical content, and holds and decays using the coding system originally devised for both co-speech gesture and sign language by Kita et al. (1998).

The paper is structured as follows. Section “Materials and Methods” provides details on our data collection as well as the annotation scheme. Section “Results” presents a statistical comparison of turn-timing in the NGT sample to the spoken languages as reported by Stivers et al. (2009). Finally, section “Discussion” discusses the methodological implications of our work.

## Materials and Methods

### The NGT Interactive Corpus

This study exploits the NGT Interactive corpus, which consists of spontaneous conversations of native NGT signers in informal settings, which have been collected, and analyzed by Merel van Zuilen, Stephen C. Levinson and Connie de Vos (Max Planck Institute for Psycholinguistics), and Onno Crasborn (Radboud University) from early 2011 onward. All data and analyses have been ethically approved by the Radboud University Ethical committee under the research program *De structuur en ontwikkeling van conversaties in gebarentaal* (De Vos and Levinson; project code ECG2012-1304-098).

The recording sessions of the NGT Interactive corpus took place in participants’ homes, at various deaf clubs, as well as a small restaurant, between participants who were long-term acquaintances and friends. These signers were also very familiar with the research assistant who recorded them, and who is a deaf native signer of NGT herself. The data therefore has the character of natural conversation. All conversations were recorded using two HD cameras from different camera angles. The data on which the present study is based features 16 signers (seven females) in one triadic and six dyadic interactions totaling 11 h and 2 min of raw video data. In one of the dyadic interactions, a third person occasionally joined the conversation, but did not participate in any of the question–answer sequences in our analysis. For this reason, this recording is treated as a dyadic interaction. All but one of the signers included in this study had acquired NGT early in life, before the age of ten and all three variants of NGT – Northern, Western, and Southern – are represented in the sample. Conversation topics ranged, unprompted by the investigators, from a work meeting regarding the write-up of a professional paper, home improvement activities, the history of the deaf club, and interpersonal relations.

The video recordings were compressed into MPEG2 format at 1920 × 1080 resolution and 25 fps. The relevant sections were then translated into written Dutch and annotated further using ELAN video annotation software (Crasborn and Sloetjes, 2008). As is customary in sign language research, each sign was glossed using a designated ID-gloss stemming from the Corpus NGT (Crasborn and de Meijer, 2012), and supplemented with novel ID-glosses whenever necessary. Non-manual signals, such as head and body movements, eyebrow movement, and eyegaze were coded in multiple independent tiers.

### Identification of Question–Answer Sequences

In order to ensure a diverse sample of question–answer sequences, we selected 30 min segments from each video file that were dense in turn transitions. In identifying these sequences, we adopted the selection criteria which were originally developed as part of the MPI Coding Scheme for Question–Response Sequences in Conversation at the MPI for Psycholinguistics (Enfield et al., 2003) and form the basis of Stivers et al. (2009). NGT polar questions are canonically marked out by raised eyebrows and a head tilt, while content questions are accompanied by a frown (Coerts, 1992). It is also syntactically possible for a content question to be formed in the absence of a wh-sign, as long as the signer uses furrowed brows. More recent work on NGT has also indicated that the brow movements associated with different question types may also be affected by paralinguistic factors, such as affect, and that these non-manual signals are therefore not a reliable cue to syntactic sentence type (De Vos et al., 2009). For these reasons, all questions in our sample were selected based on functional criteria, regardless of whether they made use of an interrogative sentence type. Specifically, we included all turns that evoked an informative answer on behalf of the addressee. Questions that were offered in reported speech, requests for physical actions, rhetorical questions, and two or more questions that were subsequently delivered in a single turn were excluded from the analysis.

Importantly, sign languages are essentially multi-modal in nature in the sense that signers do not only use their hands but also their facial expressions and body postures to express meaning at the linguistic and paralinguistic level. The non-manual components are sometimes considered as the equivalent of intonation or prosody in sign (see for instance van der Kooij et al., 2006; De Vos et al., 2009 on NGT). In the interactions we studied, a facial expression functioned on occasion as a turn on its own, for example when the combined use of a frown with a nose wrinkle and eyegaze at the addressee was taken as an open class repair initiator (similar to spoken *huh?*). Similarly, in some contexts, polar questions evoked a minimal response such as a head nod (*yes*) or a side-to-side headshake (*no*). Stivers et al. (2009) report that in spoken interactions, such visible behaviors result in faster turn transition times compared to vocal-only responses in the majority of languages in their sample. Similarly, non-manual signals in sign languages may often times persist beyond question boundaries and it is unclear at present to what extent each signal should be regarded as part of the turn at talk (De Vos et al., 2009; McCleary and de Arantes Leite, 2013). Our current analyses are therefore focused on the propositional content of the utterance as expressed by the movements of the hands. These manual movements are phonologically specified as part of the language and are most comparable to spoken words as such. Consequently, we have excluded 24 items of the original data set in which either the question or its response were solely expressed non-manually. The remaining set of functional questions were further categorized into polar questions and content questions resulting in a total data set of 190 questions, of which were 104 polar questions and 86 content questions. Overall, polar questions were thus slightly more common than content questions, as is the case in nine out of the 10 spoken language samples analyzed by Stivers et al. (2009:10588). All of the 16 signers that contributed to the corpus are represented in the sample as both questioner and answerer. The triadic conversation included 42 question answer sequences, whereas the dyadic conversations included 44, 28, 28, 23, 16, and 9 question–answer sequences, respectively.

### Movement Phase Coding

**FIGURE 1:**
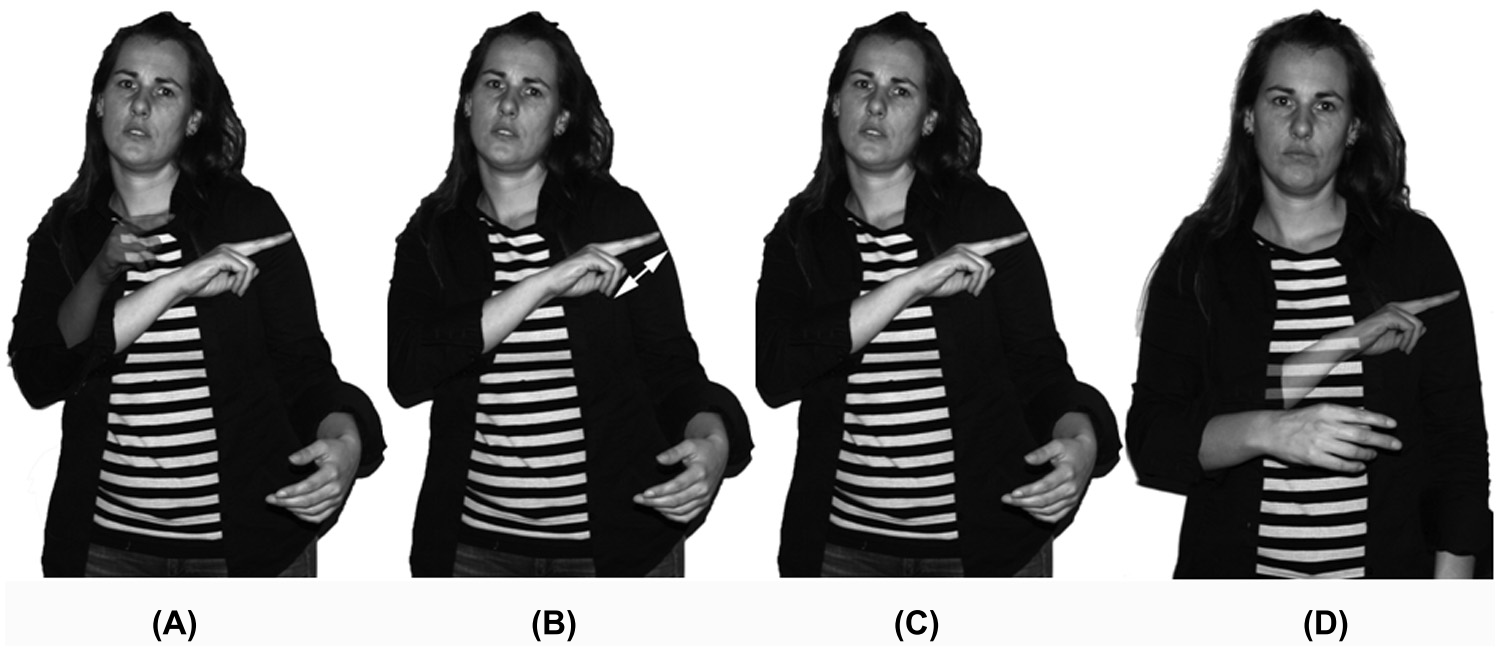
**The four gestural movement phases of the Nederlandse Gebarentaal (NGT) sign BROER ‘brother’: (A) preparation, (B) stroke, (C) hold, and (D) retraction**.

Our analysis of turn-timing is based on the coding of the various movement phases that make up a sign. Specifically, we adopted the movement phase coding developed for Sign Language of the Netherlands and co-speech gesture (Kita et al., 1998). This coding system distinguishes four movement phases for each sign: preparation, stroke, hold, and retraction. These movement phases are illustrated in **Figures 1A–D**. This figure displays all four movement phases in relation to the Dutch sign for ‘brother,’ which is produced by touching the contra-lateral upper arm twice with the middle and index fingers extended. During the preparation phase the hands move into position and the lexically-specified hand shape is selected (**Figure 1A**). The stroke most directly represents the phonological form of a sign and includes the internal movement of a sign, in this case touching the contra-lateral upper arm (**Figure 1B**). Subsequently to the stroke, a signer might hold a sign during interaction, for example to mobilize a response in their interlocutor (**Figure 1C**). Finally, the sign may be retracted and the hands move into resting position (**Figure 1D**).

Importantly, each sign is minimally associated with a single stroke, but the other three movement phases do not always occur (Kita et al., 1998). This analysis is compatible with the view that signs tend to be monosyllabic, consisting typically of a single phase (a path movement and/or a single hand-internal movement) or a repetition of a path movement or hand-internal movement (cf. Coulter, 1982; Brentari, 1998; Sandler, 1999; van der Kooij and Crasborn, 2008). The beginning and end points of each stroke were identified on the basis of the initial and final frame in which the lexically-specified hand shapes for the relevant sign were fully formed. Furthermore, for signs that include a phonologically specified internal movement, the stroke may be lengthened by a repetition of this internal movement, rather than transitioning into an utterance-final hold (cf. Perlmutter, 1992; Nespor and Sandler, 1999; Stewart, 2014). We have also observed this phenomenon our NGT data set, and in such cases only the initial inherent movement, which is lexically-specified, was included into the stroke.

For a subset of items (59 questions) a second coder, who is also a native signer of Sign Language of the Netherlands, applied the same Gesture Phase coding system. Subsequently, any items that showed discrepancies of more than two video frames were discussed, and adjusted when necessary. In a few cases these differences were based in a distinct phonological analysis of the signs that were being used and these two interpretations could not be reconciled. After these discussion sessions the overall correlations between these two coders was 0.9 for the sign-naive boundary measures and 0.98 for the stroke-to-stroke boundary measures.

### Phonetic Measures

**FIGURE 2:**
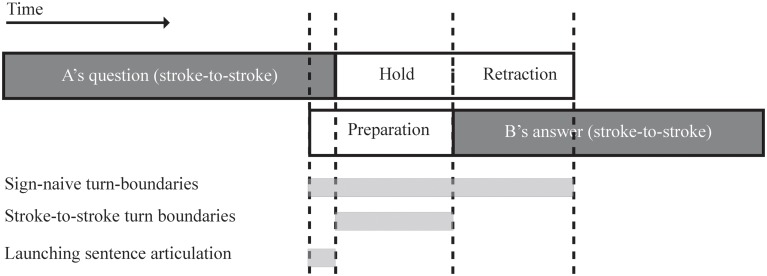
**Schematic representation of the three different phonetic measures reported in this study: sign-naive turn-boundaries, stroke-to-stroke turn boundaries, and launching sentence articulation**.

In our study we report on three phonetic measures of turn transition times based on the coding of gesture phases. The first measure looks at sign-naive turn-boundaries and includes all manual actions, that is, all movement phases that make up a signer’s utterance. The second measure looks at stroke-to-stroke turn boundaries, which run from the start of the initial stroke of a turn till the end of the turn-final stroke. For each signer, gestural movement phases from both hands were taken into account. The third and final measure calculates the offset of the addressee’s preparation phase with respect to the end of the question’s final stroke, and is called signed utterance launch. **Figure 2** illustrates each of the reported phonetic measures schematically. In section “Results,” each of these phonetic measures of signed turn-timing are compared to findings from the spoken turn-timing in cross-linguistic study presented by Stivers et al. (2009).

**FIGURE 3:**
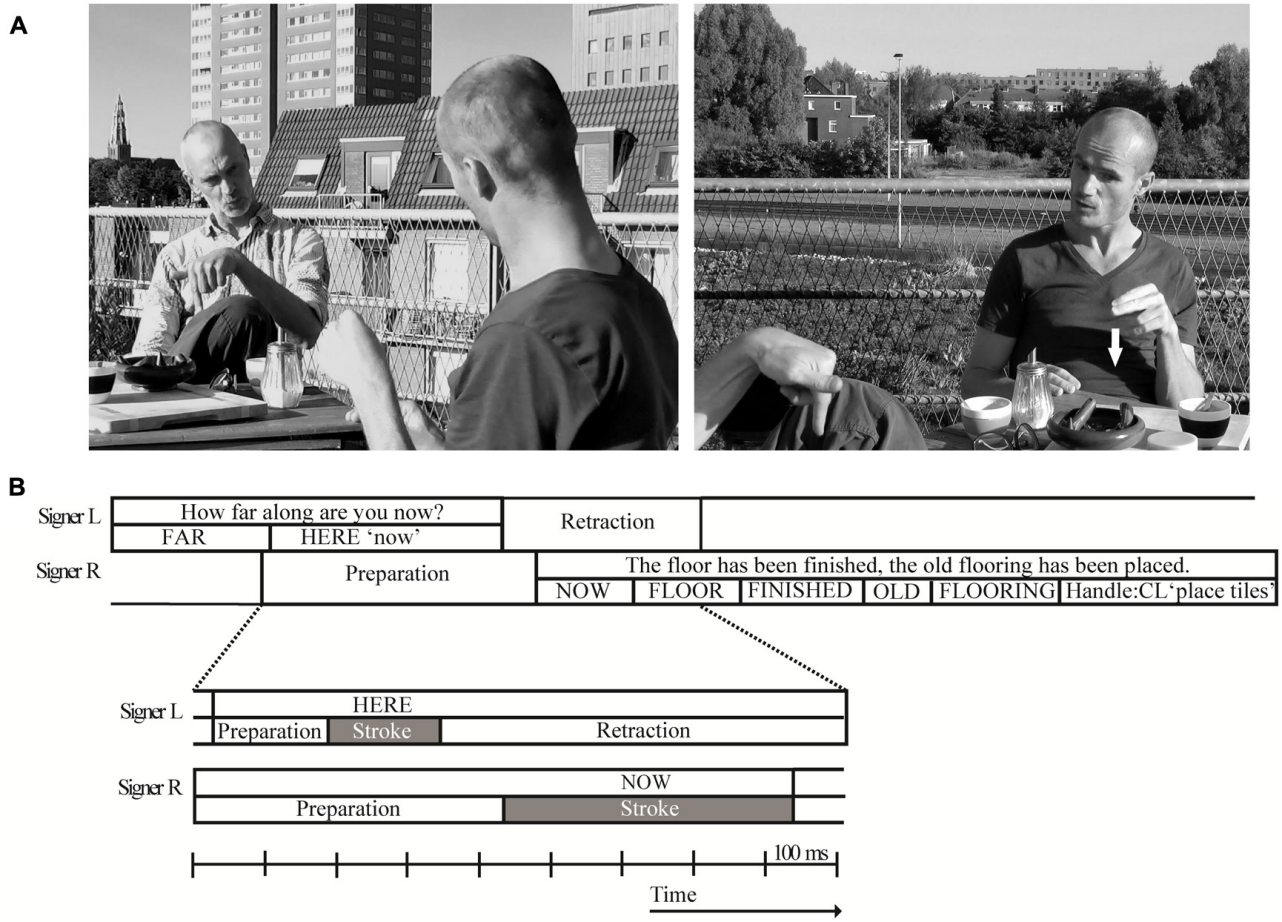
**Example of a turn transition from the NGT Interactive corpus: (A) snapshot of a turn transition; (B) scaled representation of the gestural movement phases during the turn transition**.

**Figure 3** displays an excerpt from the corpus to exemplify the three phonetic measures. This question–answer sequence stems from a conversation between two males who are close friends living in the North of the Netherlands. They are discussing remodeling activities while enjoying lunch on a roof top terrace. Directly preceding the excerpt, the signer to the viewer’s right (R) indicates his misunderstanding using a minimal repair initiator by furrowing his brows and leaning forward similar to a spoken ‘*huh?*’ (cf. Dingemanse et al., 2013). As explained above, when questions were formed without manual movements these were not included into the current analysis. In this example, each signer uses only a single hand to produce the relevant signs. At the start of the excerpt, the signer on the left (L) asks how far along signer R has progressed regarding the renovation of a particular venue. His question ends in the lexical sign HERE (Dutch: *hier*), which is formed by a downward index finger point, and co-produced with the Dutch mouth movement *nu* ‘now.’ At the end of his question, the sign HERE is immediately retracted over 430 ms without a sentence-final hold, presumably because the addressee (signer R) has already raised his hand to produce an appropriate response. Signer R’s answer starts off with the sign NOW, and he initiates the preparation of this initial sign before signer L has initiated the start of the preparation of his final sign. As a result, both signers overlap by 690 ms according to the sign-naive turn boundary measure and the launch of R’s sentence articulation has a negative value of -260 ms. According to the stroke-to-stroke measurement, however, there is a slight gap of 30 ms between both turns. This turn transition is detailed in (**Figure 3A**) which displays snapshots from both camera angels of the final frame of Signer L’s turn-final stroke. On the left-hand side there is a clear view of signer L and the end state of the sign HERE. The camera view on the right-hand side clearly shows signer R who is in the middle of the preparation phase of his turn-initial NOW; the white arrow indicates the trajectory of its stroke. The exact timing of this turn transition is illustrated by a scaled representation of the gestural movements of this turn transition in **Figure 3B**.

## Results

### The Timing of Question–Answer Sequences

**FIGURE 4:**
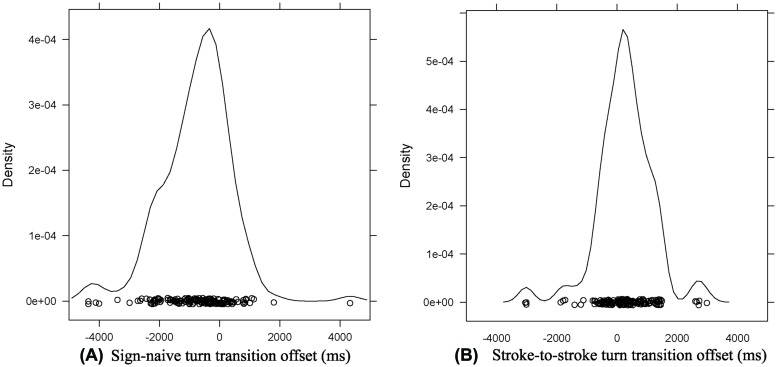
**Density plots of answer latency according to the sign-naive (A) and stroke-to-stroke (B) turn boundary measures**.

To test whether signers optimize turn-taking on the basis of stroke-to-stroke turn boundaries, we compared turn transition times in NGT with turn transition times in spoken question–answer sequences in ten languages as reported in Stivers et al. (2009). In what follows, we adopt the Floor Transfer Offset (FTO) representation used in De Ruiter et al. (2006), in which gaps are measured in positive milliseconds, overlaps in negative milliseconds. **Figure 4A** shows a density plot of turn transition offset for the overall data set according to the sign-naive turn boundary measure. The sign-naive boundary measure calculates turn-timing by including all gestural movement phases of the hands. According to this phonetic measure, the average offset of answers to questions was -812 ms, the median was -607 ms, and the mode (estimated with the density() function in R set to default parameters; R Core Team, 2014, and corresponding to the highest value in the density plot in **Figure 4A**) was -361 ms. These negative values of central tendency indicate that addressees generally start signing well before the question has fully ended. The average value of -687 ms was 6.18 SDs below the cross-linguistic average turn transition time as estimated from Stivers et al. (2009); *m* = 229 ms, SD = 168 ms). Assuming that average turn transition times across spoken languages are normally distributed, and using the data reported in Stivers et al. (2009) to estimate the parameters of this distribution, the probability of observing such a value in this distribution or lower is extremely low (*p* < 0.0001). The sign-naive turn boundary measure thus suggests that the timing of responses to questions in the visual modality deviates substantially from oral-auditory turn-taking in that it exhibits both more and more extended overlap.

**FIGURE 5:**
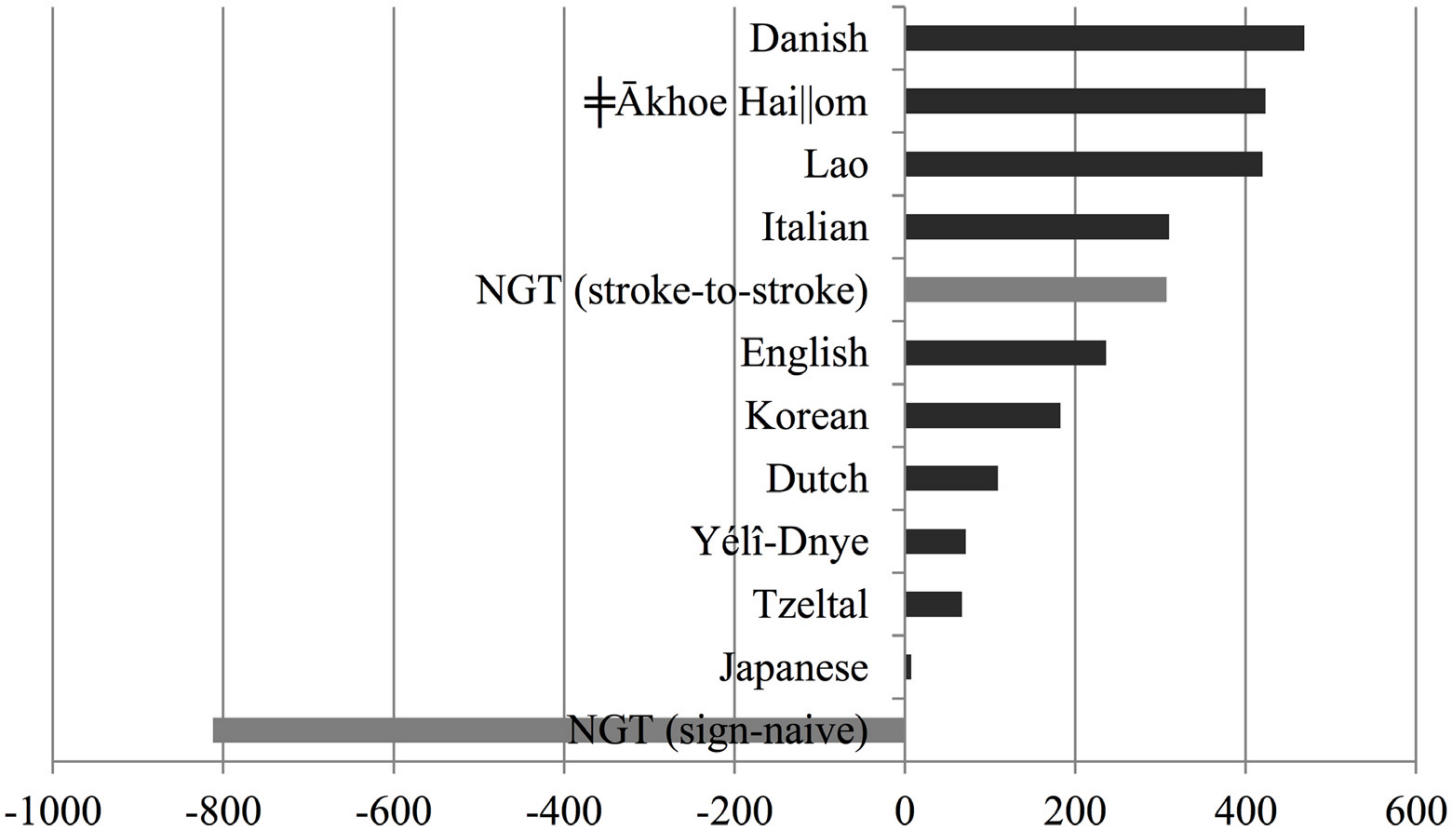
**Average turn transition times in 10 spoken languages (in dark gray, from Stivers et al., 2009) and in NGT (in light gray)**.

Secondly, we calculated turn-timing following the stroke-to-stroke boundary measure, which is based on the hypothesis that signers observe stroke-to-stroke turn boundaries. This phonetic measure calculates the offset of the answerer’s initial stroke with respect to the end of the final stroke of the questioner. **Figure 4B** shows a density plot of the stroke-to-stroke turn boundary measure. According to this measure, turn transition times in NGT now exhibit a positive gap, with an average of 307 ms, a median of 269 ms, and an estimated mode of 227 ms. The average value of 307 ms was only 0.46 SD above the cross-linguistic average turn transition time as estimated from Stivers et al. (2009). Assuming that the distribution of average turn transition times across spoken languages is normal, and using the numbers in Stivers et al. (2009), the probability of observing a value of 372 ms or higher is well above the commonly used alpha level of 2.5% for a two-tailed test (*p* = 0.32). When we exclude utterance-initial preparatory movements, and utterance-final holds and decays, turn-timing in signed interaction thus falls within range of oral-auditory turn-taking as reported by Stivers et al. (2009). This is illustrated in **Figure 5**, which shows the average turn transition times in Stivers et al. (2009) plus our two NGT measures.

### The Proportion of Overlapping and Delayed Turn Transitions

Another way of looking at signed versus spoken turn-timing is by comparing the distributions of overlapping and delayed turn transitions. Heldner (2011) previously showed that, with regard to spoken Swedish the threshold for noticeable gaps and overlaps lies at FTOs of 120 ms and -120 ms, respectively. At the moment, it is unknown whether this threshold would generalize to other spoken languages, e.g., to those that have relatively fast or slow turn transition time on average, such as Japanese or Danish, or indeed to sign language. Notwithstanding this caveat, for the sake of comparability, we here consider any turn transition offsets that exceed 120 ms as turns with a noticeable gap, and any turn transition offsets that are -120 ms or less as overlapping in order to compare the distributions of the NGT sample to the Stivers et al. (2009) study.

**FIGURE 6:**
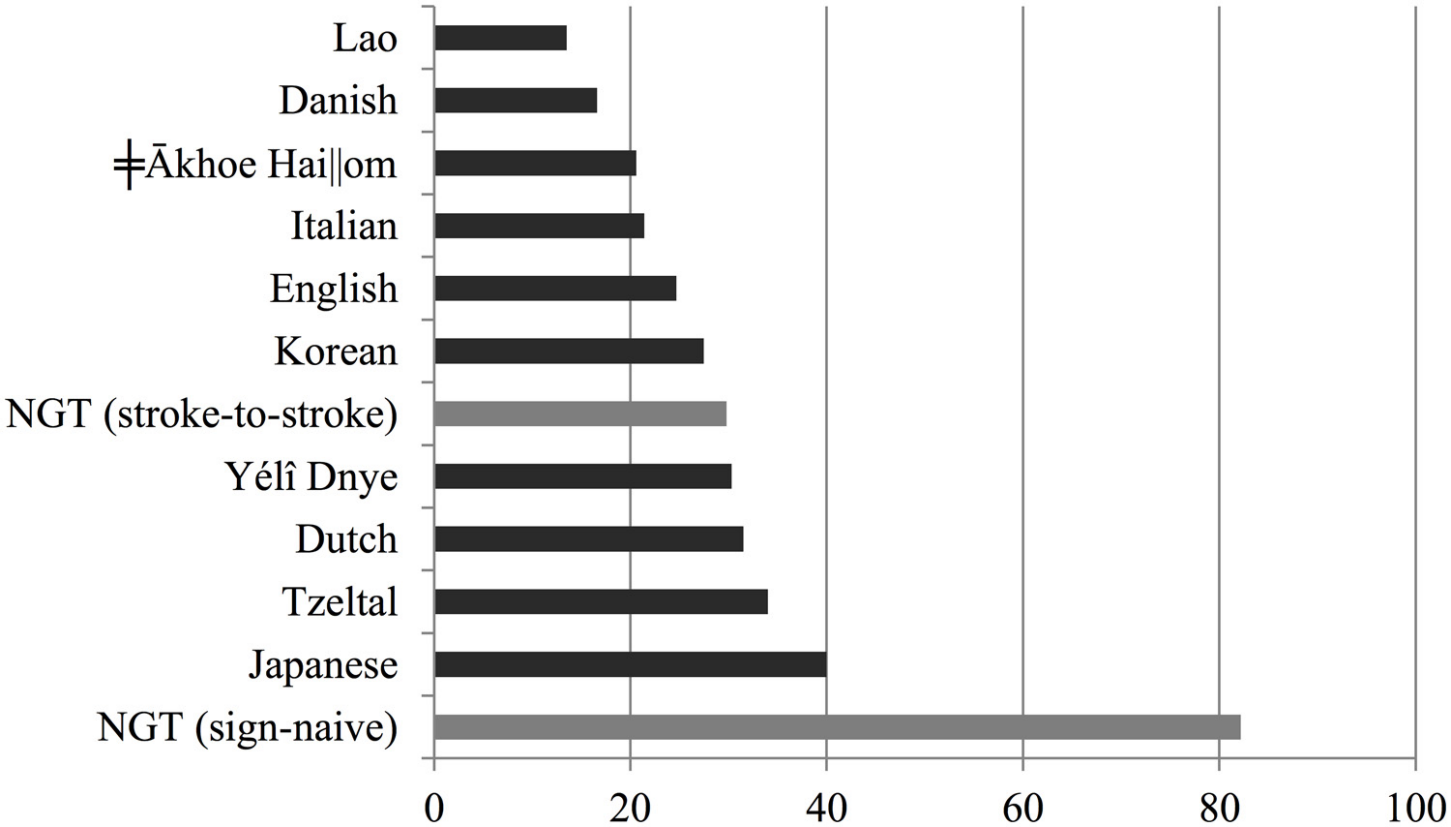
**Percentage of turn transitions overlapping 120 ms or more for 10 spoken languages (in dark gray) and in NGT (in light gray).** Non-NGT data based on Table II in Heldner (2011:519).

**Figure 6** presents an overview of the percentages of overlapping answers to questions, including the data reported in this study. As estimated by Heldner (2011) based on data from Stivers et al. (2009), the proportion of overlapping turn transitions in question–answer sequences, may range from 13.5% as reported of Lao to 40.0% as reported for spoken Japanese, with a mean of 26.01% and SD of 8.2%. When considering stroke-to-stroke boundaries, 29.8% of responses to questions come in overlap, which is within the cross-linguistic range. Assuming that the distribution of percentages of overlaps in the cross-linguistic sample is normally distributed, and using the data in Stivers et al. (2009) to estimate its parameters, the probability of observing this value or higher in such distribution is well above the commonly used alpha level of 5% (*p* = 0.28). According to the sign-naive measure, however, 82.2% of answers overlap with the respective question. This percentage clearly falls outside the cross-linguistic range for spoken languages. The probability of observing this value or a higher value in the cross-linguistic distribution is extremely low (*p* < 0.00001).

**FIGURE 7:**
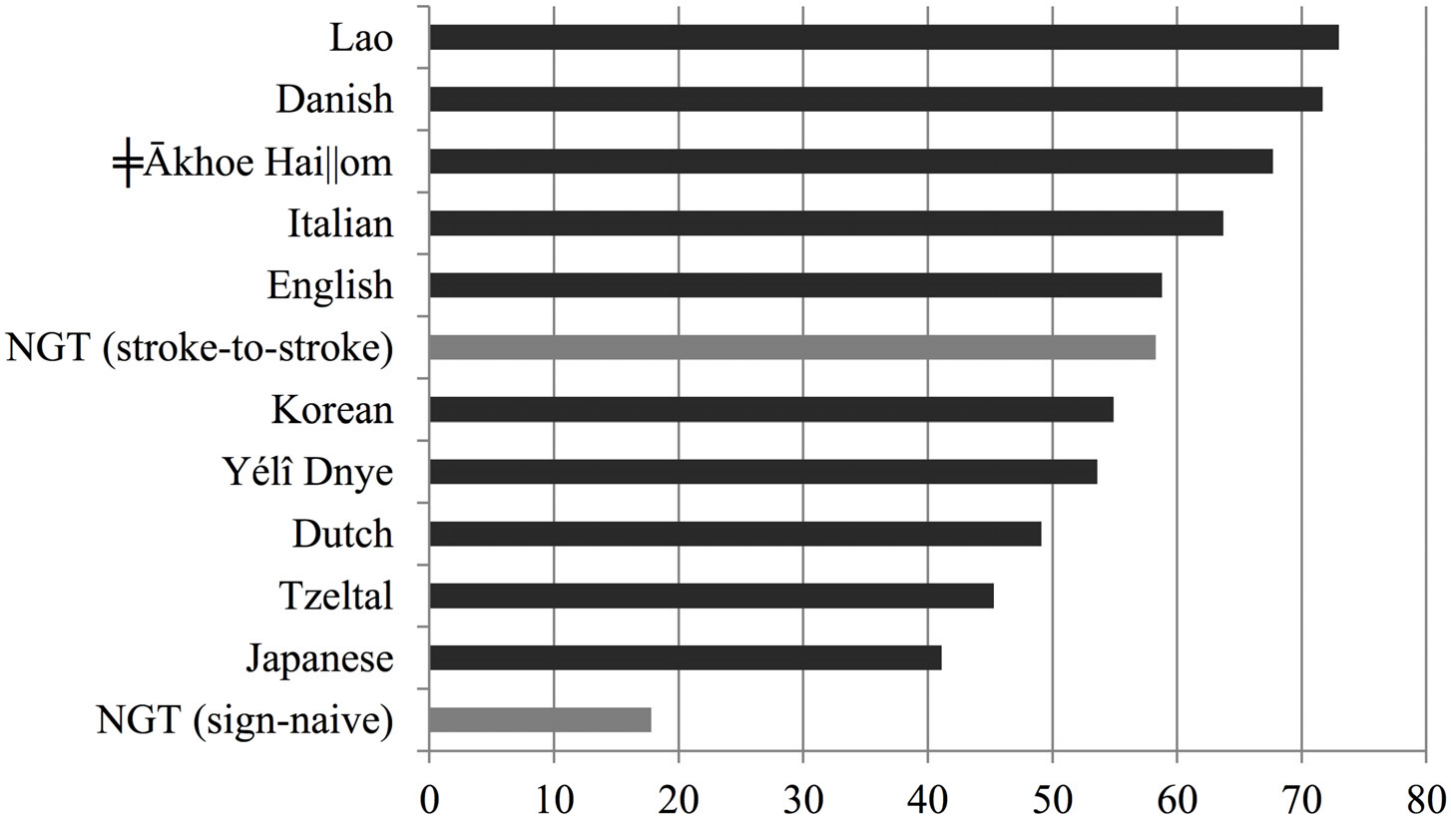
**Percentage of turn transitions with a gap of more than 120 ms for 10 spoken languages (in dark gray) and in NGT (in light gray).** Non-NGT data based on Table II in Heldner (2011:519).

**Figure 7** presents an overview of the percentages of transitions between answers and questions involving a gap of more than 120 ms, including the data reported in this study. As estimated by Heldner (2011) based on Stivers et al. (2009), the proportion of delayed turn transitions in question–answer sequences may range from 41.1% as reported of Japanese to 73.0% as reported for spoken Lao. When considering stroke-to-stroke boundaries in the sign language data, 58.3% of responses to questions had a gap of 120 ms or more; this is within the cross-linguistic expectations. Assuming that the distribution of percentages of overlaps in the cross-linguistic sample is normally distributed, the probability of observing a value of 58.3% or lower is well above 5% (*p* = 0.54). According to the sign-naive measure, however, only 17.8% of answers overlap with the respective question, which would be three times less than the spoken language which allows for the smallest number of delays in question–answer sequences. The probability of observing this value or a lower value in the cross-linguistic distribution is much lower (*p* < 0.00001).

### Launching Utterance Articulation in Sign

Unlike spoken languages, signed languages offer us the unique opportunity to examine turn preparation using non-invasive methods. In our data, the preparatory phase of utterances had an average duration of 474 ms, a median of 280 ms, and an estimated mode of 255 ms. We computed a third turn transition timing measure corresponding to the latency between the end of the final stroke of the question and the onset of the initial preparatory movements of answer articulation. Relative to the end of the final stroke of the question, response articulation in our data starts with an average latency of -86 ms, a median latency of -78 ms and a modal latency of -53 ms. The timing of preparatory movements in sign is thus slightly earlier than the initiation of pre-utterance inbreaths in answers to questions in spoken Dutch (15 ms; Torreira et al., 2015). We return to this point in the discussion section. **Table 1** summarizes all main results from the present corpus analysis.

**Table 1:** Summary of main results: mean, median, and mode for each phonetic measure of turn-boundaries (in milliseconds).

	Mean	Median	Mode
Sign-naive turn boundaries	-812	-607	-361
Stroke-to-stroke turn boundaries	307	269	227
Launching utterance articulation	-86	-78	-53

### Turn-Timing in Dyadic vs. Triadic Interactions

As explained in Section “The NGT Interactive Corpus,” one of the recordings in our corpus consisted of a triadic interaction, the other six involving dyadic conversations. Because turn-timing might differ between triadic and dyadic interactions (e.g., due to increased competition for the floor), we examined each of the timing measures presented in the previous subsections (i.e., sign-naive turn boundaries, stroke-to-stroke turn boundaries, and the launching of utterance articulation) as a function of the number of participants in the interaction. We fitted a series of mixed-effects regression models with each of the timing measures as the response, number of participants (dyadic vs. triadic) as a fixed factor, and conversation as a random factor. None of the three models yielded a statistical effect for the fixed factor number of participants (*p* > 0.1 in all three cases), indicating that the triadic conversation was not significantly different from the dyadic conversations in terms of turn-timing.

## Discussion

This study uses a corpus analysis of Sign Language of the Netherlands (NGT) in order to address the question as to what degree turn-timing in signed conversation differs from turn-timing in spoken conversation. The present study has focused on responses to questions, as questions require a timely and contingently relevant response, and could therefore serve as a baseline measure as to how much overlap or gap might be allowed in a given language. Moreover, the timing of responses to questions has been documented in a wide range of spoken languages (Stivers et al., 2009), thus allowing for a controlled comparison to turn-timing across modalities. On the basis of the corpus analysis of spontaneous interactions in NGT, we find that signed conversation exhibits a significantly greater amount of overlap than spoken conversation when we consider all hand action phases as being part of a turn, i.e., preparation, stroke, hold and retraction movements (cf. Kita et al., 1998). Interestingly, however, when we only consider the lexically-specified movement of the hands, i.e., the strokes, turn transition times in signed conversation are clearly within the cross-linguistic range reported for spoken languages, with an estimated mode of 227 ms, and with comparable amounts of gaps and overlaps. We have also found that the timing of the beginning of the preparation phase of the response relative to the end of the last stroke of the question is slightly earlier in signed conversation than that of pre-utterance inbreaths in spoken Dutch conversation, with modes of -53 ms vs. 15 ms.

As noted in the introduction, it has previously been claimed that signed conversation exhibits more overlap than spoken conversation. Coates and Sutton-Spence (2001), for instance, have argued that signers, unlike speakers, may not adhere to a one-at-a-time principle in turn-taking. Rather, they suggest that sign language users are oriented toward a collaborative floor in which more overlap is permitted and socially valued. McCleary and de Arantes Leite (2013) criticized this study for not specifying what types of turns-at-talk may legitimately be used in overlap, and for not providing precise temporal values regarding the use of the various articulators. The present study has shown that, at least in responses to questions, turn-timing in signed conversation looks remarkably similar to that of spoken conversation if we define the delineation of turns on the basis of their stroke phases only (i.e., excluding preparatory, retraction, and hold phases). The decision to consider stroke phases alone is not an arbitrary one, since strokes encode the phonological content of an utterance more directly than other hand movements. In making a comparison between spoken and signed languages, it is therefore plausible that preparatory and retraction movements in signed conversation are best seen as parallel to the pre-beginnings and post-completion elements of spoken turns (cf. Schegloff, 1987), and that TRPs are best approximated by the end of the last stroke. Experimental and qualitative research should address these issues combining the descriptive rigor of Conversation Analysis with perception experiments.

We have also seen that, using the stroke-to-stroke measures, the proportions of turn transitions with noticeable gaps and overlaps in NGT is within the same range as spoken languages according to the stroke-to-stroke boundary measure. These comparisons were carried out on the assumption that the threshold for noticeable gaps and overlaps lies at FTOs of 120 ms and -120 ms, respectively (cf. Heldner, 2011, based on data from Stivers et al., 2009). At present, however, it is unclear as to whether sign languages users are as sensitive to gaps or overlaps as speakers are. These questions could be addressed, for instance, by manipulating turn transition times in pre-recorded signed conversations.

**FIGURE 8:**
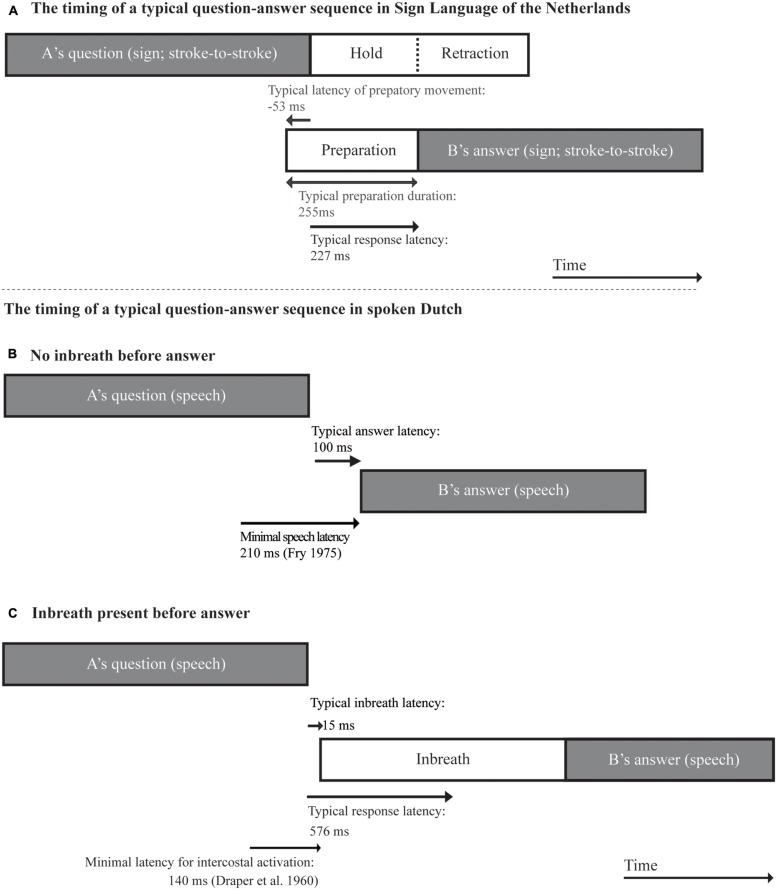
**Schematic illustration of typical time courses of: (A) a question–answer sequence in Sign Language of the Netherlands; and a question-answer sequence in spoken Dutch without (B) and with (C) a pre-utterance inbreath (based on Torreira et al., 2015)**.

In sign, the articulators are large and heavy, and reaching the articulatory targets of hand strokes from an inactive state will require more time than a vocal articulatory gesture. For this reason, the timing of preparatory hand movements preceding signed turns-at-talk may provide a crucial insight into the time course of signed utterance planning. In our question–answer sequences, we have observed that initial preparatory hand movements of responses are typically launched during the second half of the last stroke of the question, and that the preparatory phase typically ends a couple of 100 ms after the last stroke of the question. On a par with the findings of Torreira et al. (2015) for the timing of pre-utterance inbreaths in spoken conversation, our findings suggest that signers probably attend to final cues to turn-closure when launching their own articulation. If we allow for a reaction time of 200 ms (Fry, 1975), it is plausible that, in the typical case, responders initiate articulation in response to turn-final cues such as final lengthening, which, in sign language, can be manifested as an elongation, repetition, and deceleration of hand movement during the final part of the utterance (cf. Perlmutter, 1992; Nespor and Sandler, 1999; Stewart, 2014). While early cues (e.g., eyebrow movement) in the question may allow for planning the content of the response, local cues close to end of the final stroke (e.g., final lengthening) may provide a general go-ahead signal. The result of this process is a short stroke-to-stroke gap similar to the silent short gaps typically found in spoken question–answer sequences (cf. Stivers et al., 2009), and an overlap interval involving holds, retraction, and preparation phases at turn edges. **Figure 8** shows a schematic representation of typical time courses in a signed question–answer sequence based on modal values in our data (**Figure 8A**), and, for the sake of comparison, and as reported by Torreira et al. (2015) for question–answer sequences in spoken Dutch , in a spoken question–answer sequence in which the answer was produced without a preparatory inbreath (**Figure 8B**), and in a sequence in which a preparatory inbreath was produced (**Figure 8C**). Notice that, in spoken answers too, in line with our findings for sign language, the typical onset of the physical response, in the form of a preparatory inbreath, or of speech proper in answers not preceded by an inbreath, typically starts briefly after the end of the question.

The time course of turn production critically depends on the perception and comprehension of the preceding turn (Levinson, 2013). Recent work suggests that sign-perceivers use early preparatory movements to predict the content of an upcoming sign, resulting in relatively early N400 effects in online signed sentence comprehension (Hosemann et al., 2013). This phenomenon appears to be afforded by the fact that sign languages exhibit a phonological structure already partly visible in the preparatory movements, thus enabling an early incremental and predictive processing of signs. While pre-utterance inbreaths may indicate the preparation for onset of a spoken turn, pre-utterance signals in spoken language (e.g., lip position) may only offer co-articulatory information about the first segment of the lexical content of an upcoming utterance. It is true that speakers may thus use bodily visible behaviors to enable smooth turn transitions (Mondada, 2007; Ford et al., 2012; Oloff, 2013), but it is unclear at present how routinely these precede vocalization.

A related issue concerns the types of non-manual signals that might enable the accurate projection of TRPs by sign-perceivers. Reaction time experiments are currently being run to establish sign-perceivers’ sensitivity to TRPs as defined by stroke-to-stroke turn boundaries (Casillas et al., submitted). That study also aims to identify the visual information signers rely on to determine utterance boundaries online, on the basis of linguistic annotations of the visible cues in this additional data set. On a par with previous work on spoken languages (Ford and Thompson, 1996; Local and Walker, 2012; Torreira et al., 2015), we hypothesize that in addition to lexical content and syntax (De Ruiter et al., 2006; Magyari and De Ruiter, 2012), phonetic and prosodic markers such as signing speed or height (Wilbur, 2009; Russell et al., 2011), as well as visual intonation on the face may play a role (Reilly et al., 1990; Nespor and Sandler, 1999; Fenlon et al., 2007; Dachkovsky and Sandler, 2009; Dachkovsky et al., 2013) in the online prediction of stroke-to-stroke turn boundaries.

This paper has centered on question–answer sequences within a relatively limited data set. Our findings are in accordance with the hypothesis that there may be a single turn-taking system underlying both signed and spoken interactions (Levinson, 2013). If this prediction is borne out by further research, it will extend the discovery that sign languages share all the core features of human language including the domain of communicative turn-taking. Our findings are also consistent with the view that the turn-taking system may be a core part of human communicative ethology, the foundation to language itself. If so, we would expect turn-timing in deaf communities to vary within the range of differences we find across spoken language communities. Of particular interest in this regard are sign languages which have emerged within recent generations such as home sign systems (Goldin-Meadow, 2003), signed contact pidgins (Byun et al., 2014), emerging sign languages (Meir et al., 2010) and rural signing varieties (Zeshan and De Vos, 2012; De Vos and Pfau, 2015). Even though these signing communities have limited time depths, they may follow the same turn-taking principles as other spoken and signed languages. At any rate, we believe that the delineation of turns on the basis of stroke-to-stroke turn boundaries offers a critical tool in the analysis of turn-timing in sign. Preliminary investigations indeed indicate that such an analysis of turn-timing can be instructive to differentiate various types of sequences of consecutive turns to repair misunderstanding in the interactions of signers who do not know a common sign language (Byun et al., 2014; Dingemanse et al., 2014).

In sum, the observed patterns in signed turn-timing in NGT are within the range observed for spoken languages in terms of response latency to questions once we exclude preparatory movements from turn beginnings and retraction phases from turn ends. Moreover, unlike previously argued by Coates and Sutton-Spence (2001), there is now both qualitative (McIlvenny, 1995; Mesch, 2001; McCleary and de Arantes Leite, 2013; Groeber and Pochon-Berger, 2014) and quantitative evidence that sign language users orient to a one-at-a-time principle in taking turns. All in all, our study is consistent with the view that, despite the potential differences between the visual and acoustic language modalities, spoken and signed turn-taking may share more features than has previously been suggested. Further research should center on the question as to what extent the psycholinguistic processes and time course of turn-perception and production in sign versus speech might display similarities as well as differences due to the affordances of each natural language modality.

## Conflict of Interest Statement

The authors declare that the research was conducted in the absence of any commercial or financial relationships that could be construed as a potential conflict of interest.
